# Supplementary material for: Obesity differs from diabetes mellitus in antibody and T-cell responses post-COVID-19 recovery
Source: Clin Exp Immunol. 2024 Apr 20;218(1):78–92. doi: 10.1093/cei/uxae030 (PMC11404124; doi:10.1093/cei/uxae030)
Supplement: uxae030_suppl_Supplementary_Materials [file uxae030_suppl_supplementary_materials.pdf]

Supporting Information

Obesity Differs from Diabetes Mellitus in Antibody and T Cell Responses Post COVID-19 Recovery

Mohammad Ali(1,2,3,4,10), Stephanie Longet(5), Isabel Neale(1,2,3), Patpong Rongkard(1,2,3), Forhad Uddin Hassan Chowdhury(6), Jennifer Hill(1,2,3), Anthony Brown(1), Stephen Laidlaw(5), Tom Tipton(5), Ashraful Hoque(7), Nazia Hassan(8), Carl-Philipp Hackstein(1,9), Sandra Adele(1,2,3), Hossain Delowar Akther (1,9), Priyanka Abraham(1,2,3), Shrebash Paul(8), Md Matiur Rahman(10), Md Masum Alam(10), Shamima Parvin(11), Forhadul Hoque Mollah(10), Md Mozammel Hoque(10), Shona C Moore(12), Subrata K Biswas(10, 14), Lance Turtle(12), Thushan I de Silva(13), Ane Ogbe(1), John Frater(1), Eleanor Barnes(1,2,15), Adriana Tomic (16, 17, 18), Miles W Carroll(5), Paul Klenerman(1,2,9,15), Barbara Kronsteiner(1,2,3), Fazle Rabbi Chowdhury(3,8), Susanna J Dunachie(1,2,3,15)

[1] Peter Medawar Building for Pathogen Research, Nuffield Department of Clinical Medicine, University of Oxford, Oxford, UK
[2] Centre for Global Health Research, Nuffield Department of Clinical Medicine, University of Oxford, Oxford, UK
[3] Mahidol-Oxford Tropical Medicine Research Unit, Mahidol University, Bangkok, Thailand
[4] Directorate General of Health Services, Dhaka, Bangladesh
[5] Wellcome Centre for Human Genetics, Nuffield Department of Medicine, University of Oxford, Oxford, UK
[6] Department of Internal Medicine, Dhaka Medical College, Dhaka, Bangladesh
[7] Department of Transfusion Medicine, Sheikh Hasina National Burn & Plastics Surgery Institute, Dhaka, Bangladesh
[8] Department of Internal Medicine, Bangabandhu Sheikh Mujib Medical University, Dhaka, Bangladesh
[9] Translational Gastroenterology Unit, Nuffield Department of Clinical Medicine, University of Oxford, Oxford, UK
[10] Department of Biochemistry and Molecular Biology, Bangabandhu Sheikh Mujib Medical University, Dhaka, Bangladesh
[11] Department of Biochemistry and Molecular Biology, Mugda Medical College, Dhaka, Bangladesh
[12] Tropical and Infectious Disease Unit, Liverpool University Hospitals NHS Foundation Trust, Member of Liverpool Health Partners, Liverpool, UK
[13] Department of Infection, Immunity and Cardiovascular Disease, University of Sheffield, Sheffield, UK
[14] Department of Molecular and Cell Biology, University of Connecticut, Storrs, Connecticut, USA
[15] NIHR Oxford Biomedical Research Centre, Oxford University Hospitals NHS Foundation Trust, Oxford, UK
[16] National Emerging Infectious Diseases Laboratories, Boston University, USA
[17] Department of Microbiology, Boston University School of Medicine, USA
[18] Department of Biomedical Engineering, Boston University, Boston, MA, USA

Contact Info:

Susanna Dunachie, Peter Medawar Building for Pathogen Research, University of Oxford, OX1 3SY, Oxford, UK. Email: susie.dunachie@ndm.ox.ac.uk

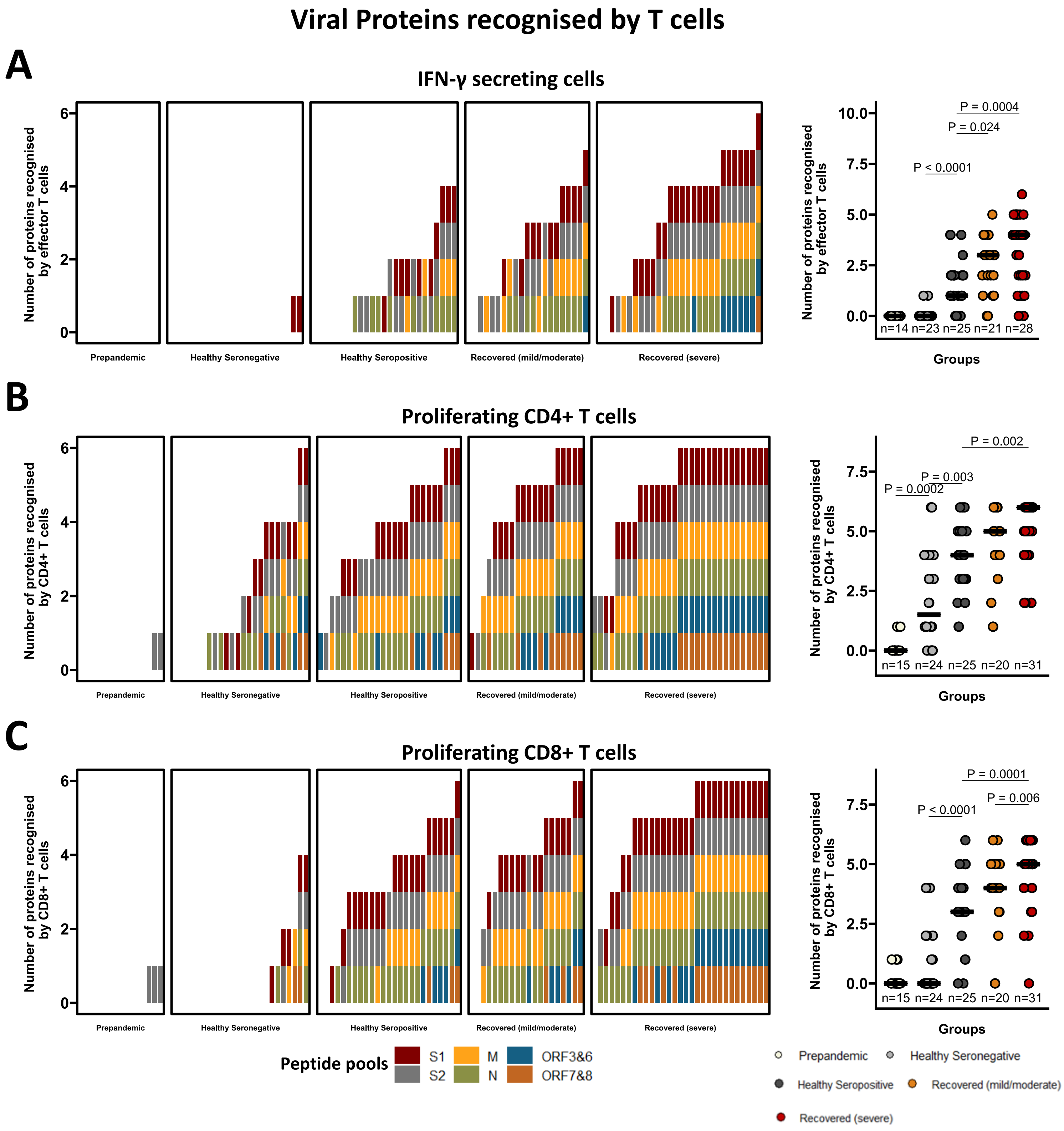

Figure S1: The breadth of T cell responses to SARS-CoV-2 was higher in patients who recovered from severe COVID-19.

Barplots on the left display the number of viral proteins recognised in individuals across groups (coloured by specificity) by (A) ex vivo IFN-γ ELISpot assay, (B) CD4+ T cell proliferation, (C) CD8+ T cell proliferation. Dot plots on the right show the comparison of the number of viral proteins recognised among groups by corresponding assays.

A two-tailed Wilcoxon rank-sum test was used to compare between the groups (without correction for multiple testing), p values are shown on the top of the dot plots The number of individuals (n) evaluated per assay is displayed at the bottom of the corresponding dot plots, while horizontal bars represent the medians.

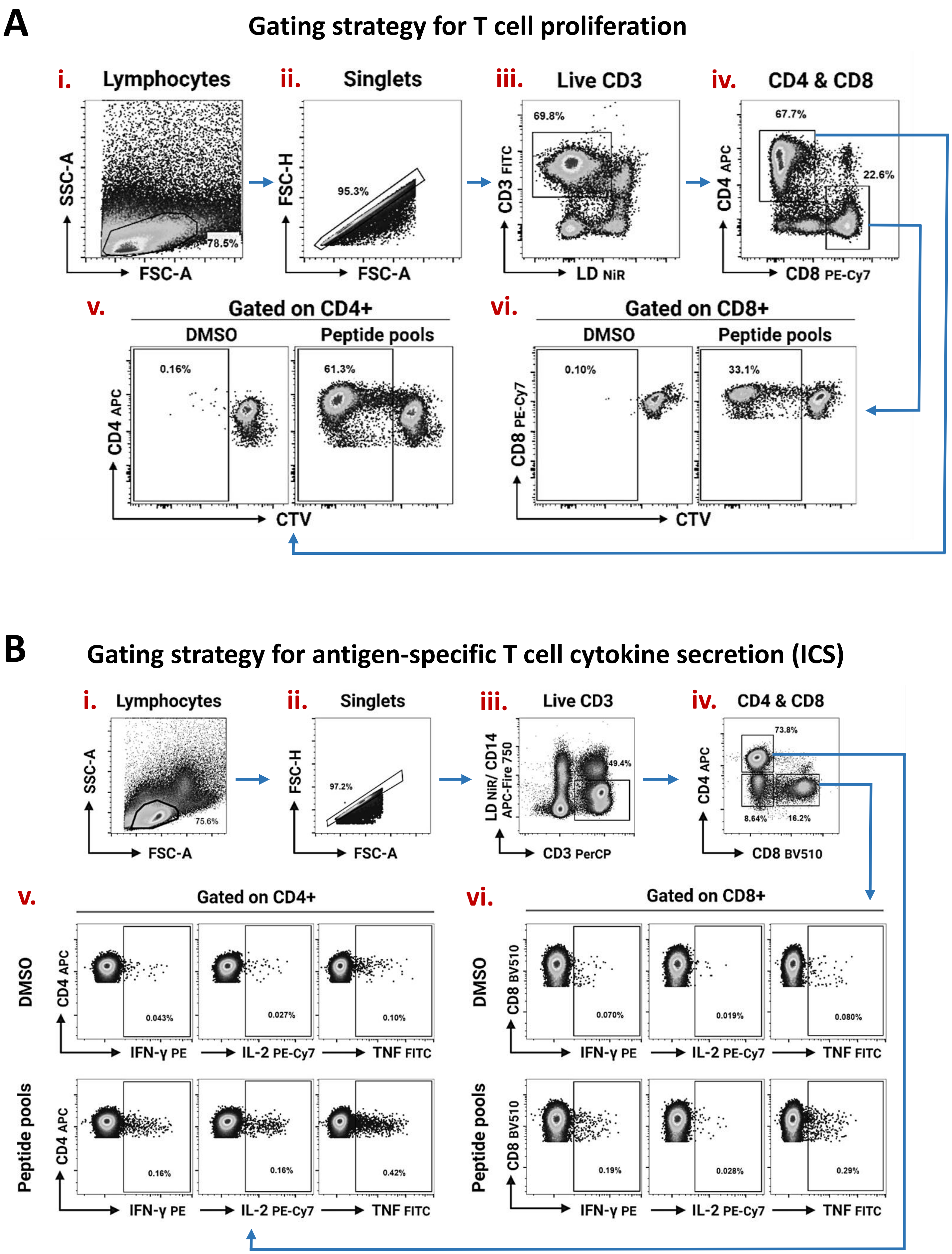
A
Gating strategy for T cell proliferation
i. Lymphocytes
SSC-A
FSC-A
78.5%
ii. Singlets
FSC-H
FSC-A
95.3%
iii. Live CD3
CD3 FITC
LD NiR
69.8%
iv. CD4 & CD8
CD4 APC
CD8 PE-Cy7
67.7%
22.6%
v. Gated on CD4+
DMSO
Peptide pools
CD4 APC
CTV
0.16%
61.3%
vi. Gated on CD8+
DMSO
Peptide pools
CD8 PE-Cy7
CTV
0.10%
33.1%
B
Gating strategy for antigen-specific T cell cytokine secretion (ICS)
i. Lymphocytes
SSC-A
FSC-A
75.6%
ii. Singlets
FSC-H
FSC-A
97.2%
iii. Live CD3
LD NiR/ CD14 APC-Fire 750
CD3 PerCP
49.4%
iv. CD4 & CD8
CD4 APC
CD8 BV510
73.8%
8.64%
16.2%
v. Gated on CD4+
DMSO
Peptide pools
CD4 APC
IFN-γ PE
IL-2 PE-Cy7
TNF FITC
0.043%
0.027%
0.10%
0.16%
0.16%
0.42%
vi. Gated on CD8+
DMSO
Peptide pools
CD8 BV510
IFN-γ PE
IL-2 PE-Cy7
TNF FITC
0.070%
0.019%
0.080%
0.19%
0.028%
0.29%

Figure S2: Gating strategy for T cell proliferation and intracellular cytokine staining (ICS).

(A) For T cell proliferation assays, lymphocytes were gated using forward and side scatter area (FSC-A and SSC-A) parameters, followed by single cell gating on FSC-height (H) and area (A). Live T cells were then gated (LD-NiR low CD3+) and T cell subsets were identified (CD4+CD8- and CD8+CD4-) using CD4+ APC and CD8+ PE-Cy7. Within the CD4+ and the CD8+ T cell gate proliferating cells were identified by gating on cells with reduced CTV (CellTraceTM Violet) fluorescence intensity.

(B) For ICS assays lymphocytes were gated using forward and side scatter area (FSC-A and SSC-A) parameters, followed by single cell gating on FSC-height (H) and area (A). Live CD3+ T cells were gated based on exclusion of dead cells (LD-NiR) and monocytes (CD14 APC Fire-750) as well as positivity for CD3 PerCP. T cell subsets were identified based on staining for CD4 APC and CD8 BV510 respectively and expression of cytokines (IFN-γ, TNF, IL-2) was then identified in the CD4+CD8- gate as well as the CD8+CD4- gate. Representative gating is shown for the DMSO negative control and the reactive peptide pools.

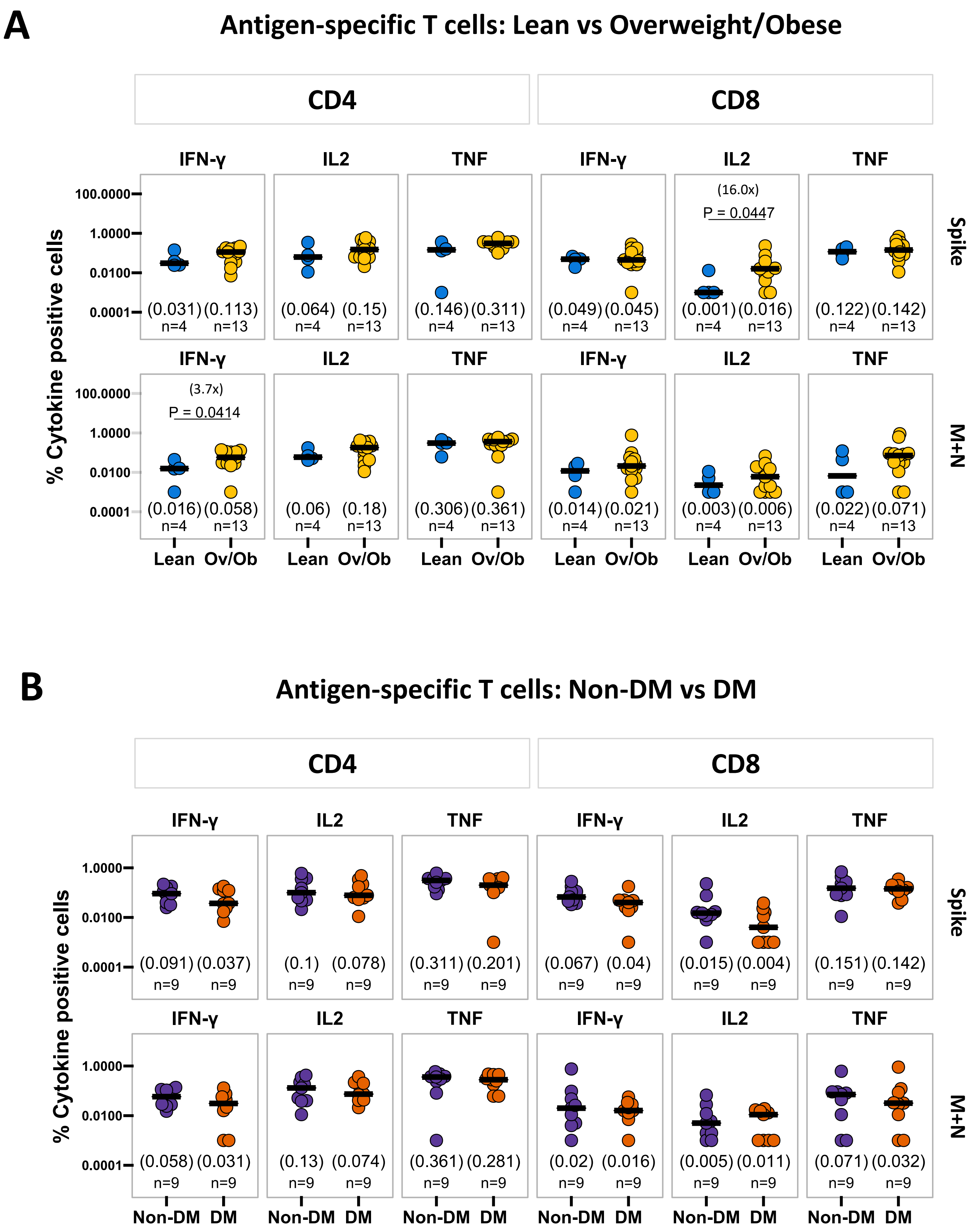
A
Antigen-specific T cells: Lean vs Overweight/Obese
CD4
CD8
IFN-γ
IL2
TNF
% Cytokine positive cells
100.0000
1.0000
0.0100
0.0001
(16.0x)
P = 0.0447
(3.7x)
P = 0.0414
(0.031) (0.113)
(0.064) (0.15)
(0.146) (0.311)
(0.049) (0.045)
(0.001) (0.016)
(0.122) (0.142)
(0.016) (0.058)
(0.06) (0.18)
(0.306) (0.361)
(0.014) (0.021)
(0.003) (0.006)
(0.022) (0.071)
n=4 n=13
Lean Ov/Ob
Spike
M+N
B
Antigen-specific T cells: Non-DM vs DM
CD4
CD8
IFN-γ
IL2
TNF
% Cytokine positive cells
1.0000
0.0100
0.0001
(0.091) (0.037)
(0.1) (0.078)
(0.311) (0.201)
(0.067) (0.04)
(0.015) (0.004)
(0.151) (0.142)
(0.058) (0.031)
(0.13) (0.074)
(0.361) (0.281)
(0.02) (0.016)
(0.005) (0.011)
(0.071) (0.032)
n=9 n=9
Non-DM DM
Spike
M+N

Figure S3: Overweight/obese status is associated with increased T cell cytokine responses to SARS-CoV-2 after recovery, but the cytokine responses are similar between diabetic and non-diabetic recovered patients.

Comparison of cytokine responses (IFN-γ, IL-2 and TNF) by $CD4^+$ and $CD8^+$ T cells in response to SARS-CoV2 spike (top panel) and M+N pools (bottom panel) between (A) lean and overweight/obese (Ov/Ob), (B) non-diabetic (Non-DM) and diabetic (DM) recovered COVID-19 patients.

A two-tailed Wilcoxon rank-sum test was used to compare between the groups (without correction for multiple testing), with fold changes in brackets. P values are displayed in case of significant differences. The number of individuals (n) evaluated per assay is displayed at the bottom of the corresponding dot plots. Horizontal bars represent the medians, and the median values are shown in brackets immediately above the number of individuals (n) in each column.

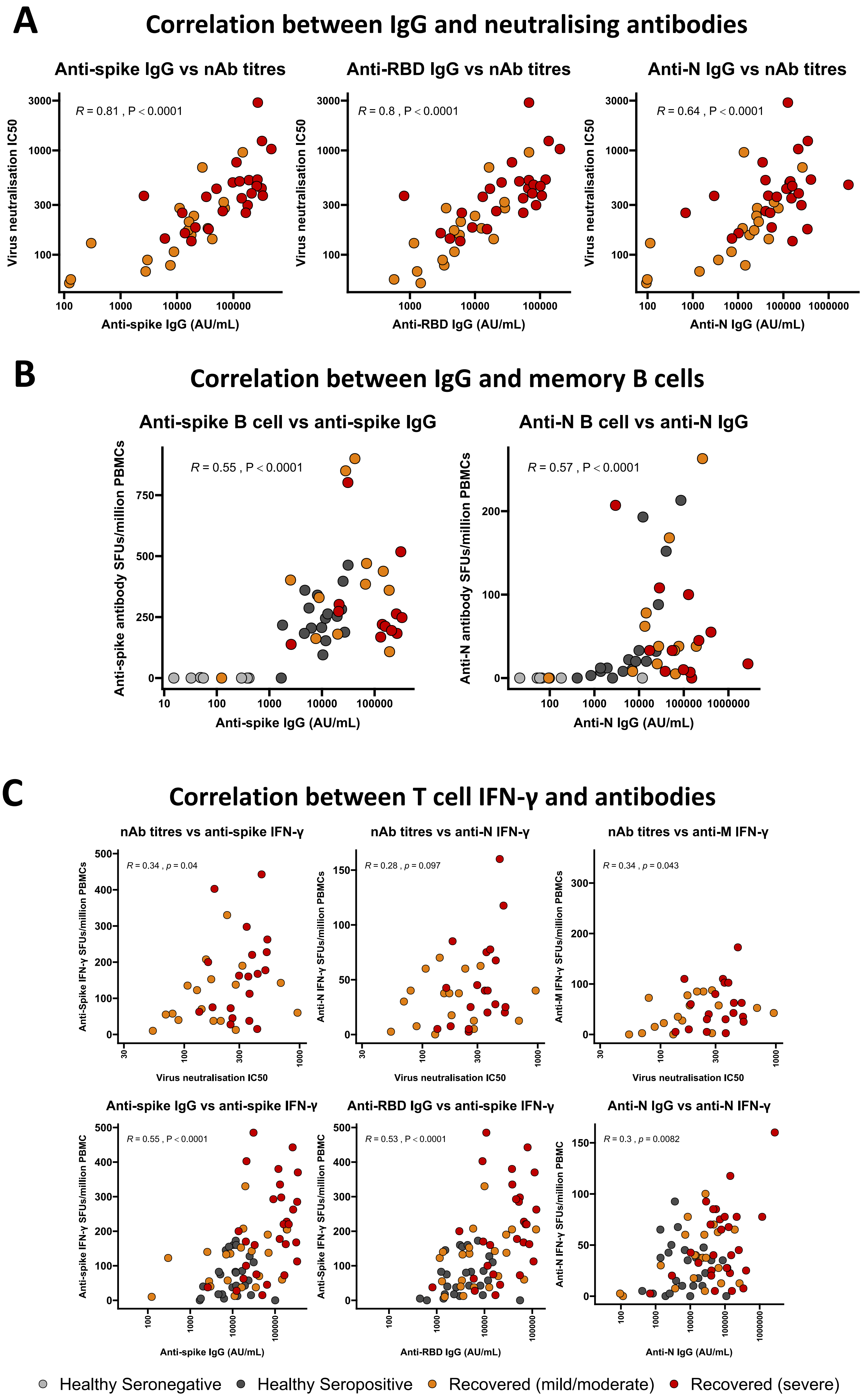
A
Correlation between IgG and neutralising antibodies
Anti-spike IgG vs nAb titres
R = 0.81 , P < 0.0001
Virus neutralisation IC50
Anti-spike IgG (AU/mL)
Anti-RBD IgG vs nAb titres
R = 0.8 , P < 0.0001
Anti-RBD IgG (AU/mL)
Anti-N IgG vs nAb titres
R = 0.64 , P < 0.0001
Anti-N IgG (AU/mL)
B
Correlation between IgG and memory B cells
Anti-spike B cell vs anti-spike IgG
R = 0.55 , P < 0.0001
Anti-spike antibody SFUs/million PBMCs
Anti-spike IgG (AU/mL)
Anti-N B cell vs anti-N IgG
R = 0.57 , P < 0.0001
Anti-N antibody SFUs/million PBMCs
Anti-N IgG (AU/mL)
C
Correlation between T cell IFN-γ and antibodies
nAb titres vs anti-spike IFN-γ
R = 0.34 , p = 0.04
Anti-Spike IFN-γ SFUs/million PBMCs
Virus neutralisation IC50
nAb titres vs anti-N IFN-γ
R = 0.28 , p = 0.097
Anti-N IFN-γ SFUs/million PBMCs
nAb titres vs anti-M IFN-γ
R = 0.34 , p = 0.043
Anti-M IFN-γ SFUs/million PBMCs
Anti-spike IgG vs anti-spike IFN-γ
R = 0.55 , P < 0.0001
Anti-spike IFN-γ SFUs/million PBMC
Anti-spike IgG (AU/mL)
Anti-RBD IgG vs anti-spike IFN-γ
R = 0.53 , P < 0.0001
Anti-Spike IFN-γ SFUs/million PBMC
Anti-RBD IgG (AU/mL)
Anti-N IgG vs anti-N IFN-γ
R = 0.3 , p = 0.0082
Anti-N IFN-γ SFUs/million PBMC
Anti-N IgG (AU/mL)
Healthy Seronegative
Healthy Seropositive
Recovered (mild/moderate)
Recovered (severe)

Figure S4: Correlation between antibody, B cell and T cell responses to SARS-CoV-2.

(A) Correlation of neutralising antibody (nAb) titers with IgG responses to SARS-CoV-2 spike, receptor binding domain (RBD), and nucleoprotein (N) in recovered COVID-19 patients.

(B) Correlation between IgG and memory B cell responses to SARS-CoV-2 spike (left) and N (right) in healthy seronegative and seropositive controls and recovered patients.

(C) Correlation of IFN-γ ELISpot responses with IgG and nAb titers in healthy seropositive controls and recovered patients.

Spearman's correlation coefficient (R) and p-values are shown at the top of each plot.

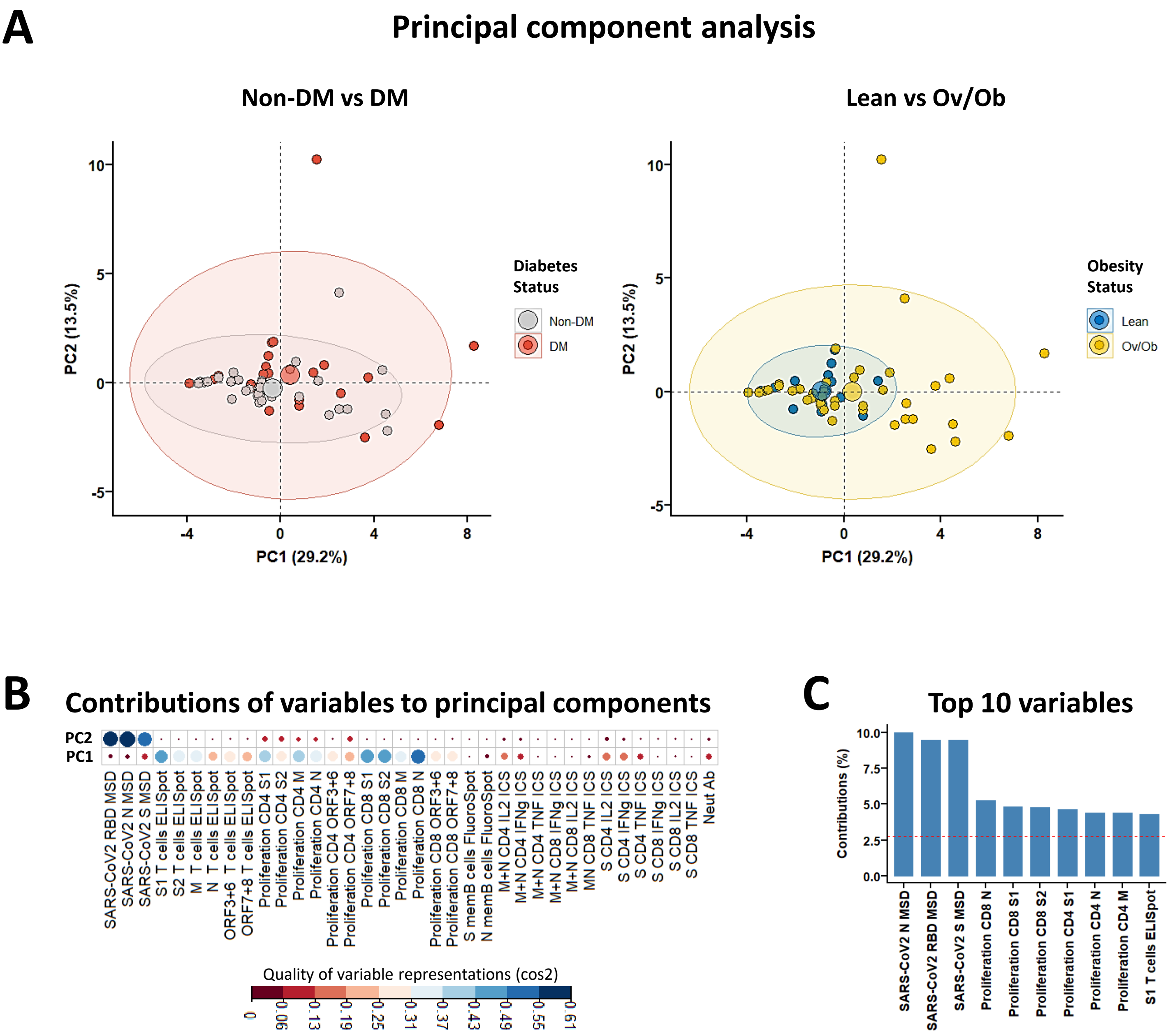

Figure S5: Principal component analysis with integrated immunological data in recovered COVID-19 patients.

(A) PCA plot representing integrated immunological data, grouped based on the diabetes (left) and obesity (right) status. Percentage indicates the variance explained by the principal component (PC).

(B) Quality of variable representations (colour-coded, cos2) and contributions of variables to principal components 1 and 2 (size of the circle).

(C) Top 10 variables and their contribution to PC 1 and 2.

A Antibody responses in recovery: Lean vs Overweight/ Obese across disease severity

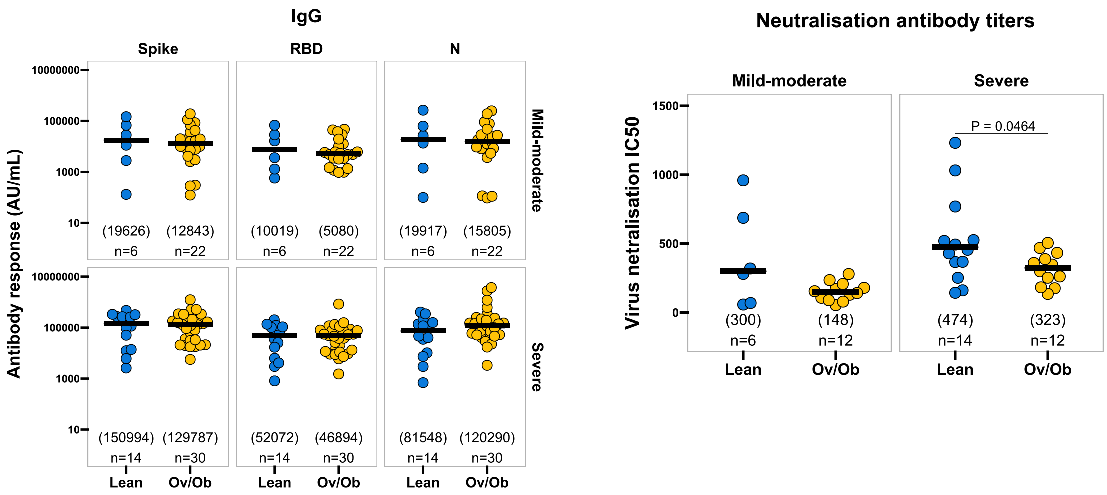

B IFN-γ ELISpot responses in recovery: Lean vs Overweight/ Obese across disease severity

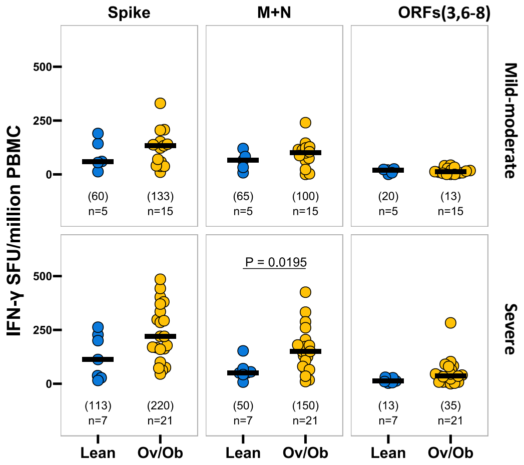

C T cell proliferation in recovery: Lean vs Overweight/ Obese across disease severity

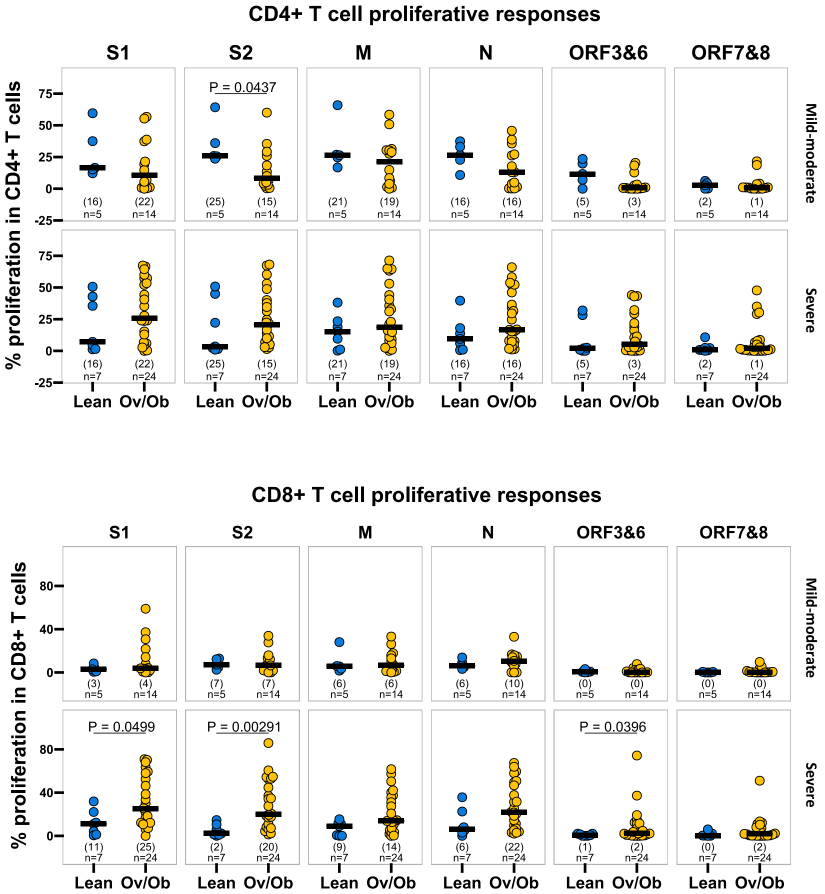

Figure S6: Antibody and T cell responses in lean and overweight/obese individuals following recovery from mild-moderate and severe disease

(A) Comparison of SARS-CoV-2 spike, RBD, N -specific IgG responses and neutralising antibody titers in lean (BMI = 18.5 – 22.9 kg/m^2) and overweight/obese (BMI ≥ 23 kg/m^2) individuals, who recovered from mild-moderate and severe disease. IgG responses and neutralising antibody titers are measured by multiplexed MSD immunoassays and Focus reduction neutralisation (FRNT) assay, respectively, and data are shown in arbitrary units (AU)/mL and IC50, respectively.

(B) Comparison of IFN-γ ELISpot responses to SARS-CoV-2 spike (summed responses to S1 and S2 peptide pools), M+N (summed responses to M and N pools), and ORFs (summed responses to ORF3, 6-8) from cryopreserved PBMCs in lean (BMI = 18.5 – 22.9 kg/m^2) and overweight/obese (BMI ≥ 23 kg/m^2) individuals in recovery from mild-moderate and severe disease. Data are shown in IFN-γ spot-forming units (SFU)/million PBMC.

(C) Comparison of the relative frequency of $CD4^+$ (top panels) and $CD8^+$ (bottom panels) T cells proliferating to individual peptide pools S1, S2, M, N, ORF3&6, ORF7&8, assessed by flow cytometry (gating strategy shown in figure S2A) from cryopreserved PBMC, in lean (BMI = 18.5 – 22.9 kg/m^2) and overweight/obese (BMI ≥ 23 kg/m^2) individuals, who recovered from mild-moderate and severe disease.

A two-tailed Wilcoxon rank-sum test was used to compare between the groups (without correction for multiple testing), and the p values are shown on the top of the dot plots, in case of significant differences. The number of individuals (n) evaluated per assay is displayed at the bottom of the corresponding dot plots. Horizontal bars represent the medians, and the median values are shown in brackets immediately above the number of individuals (n) in each column.

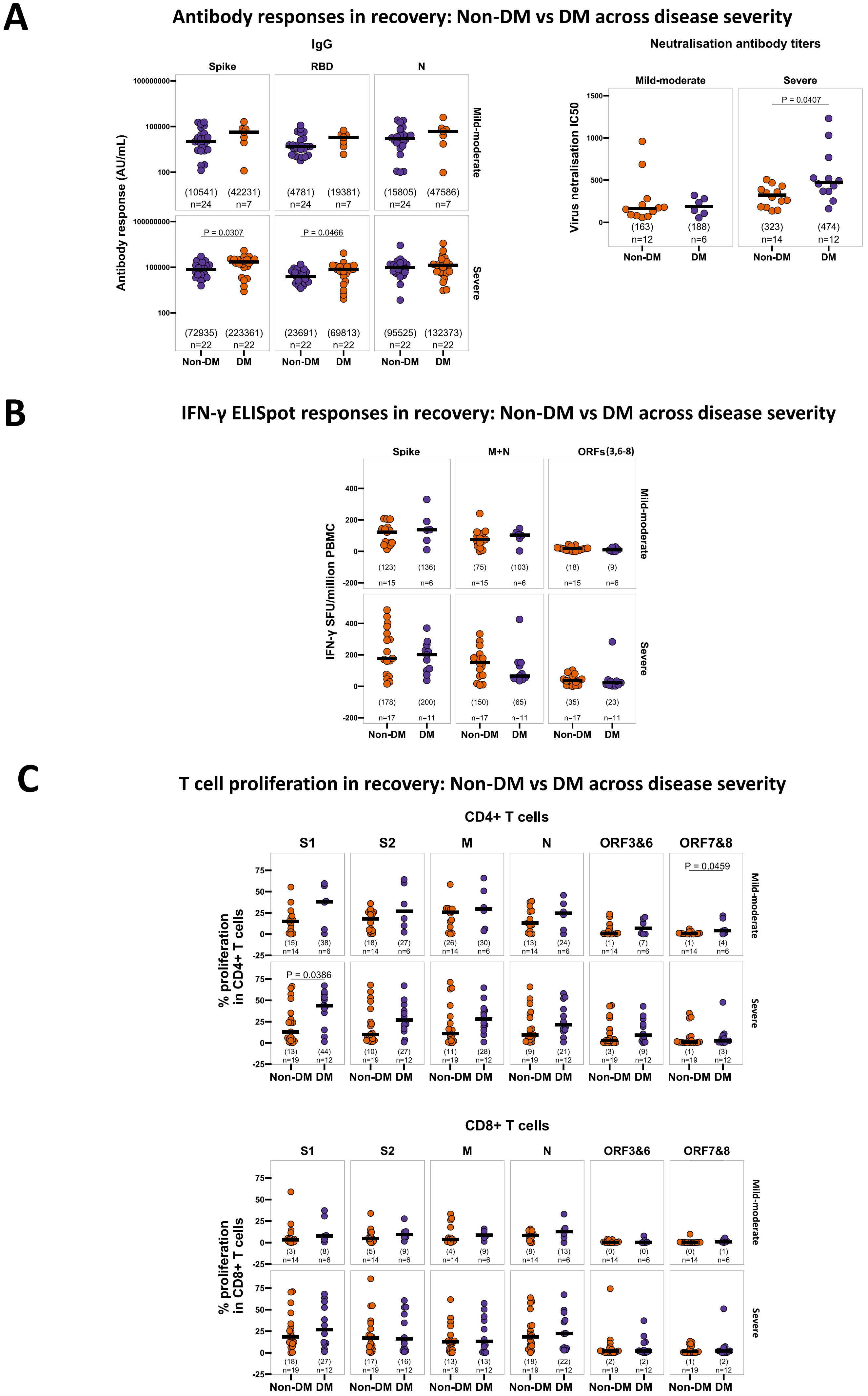
A
Antibody responses in recovery: Non-DM vs DM across disease severity
IgG
Spike
RBD
N
Mild-moderate
Severe
Antibody response (AU/mL)
100000000
100000
100
(10541) n=24
(42231) n=7
(4781) n=24
(19381) n=7
(15805) n=24
(47586) n=7
P = 0.0307
P = 0.0466
(72935) n=22
(223361) n=22
(23691) n=22
(69813) n=22
(95525) n=22
(132373) n=22
Non-DM
DM
Neutralisation antibody titers
Mild-moderate
Severe
Virus netralisation IC50
1500
1000
500
0
P = 0.0407
(163) n=12
(188) n=6
(323) n=14
(474) n=12
B
IFN-γ ELISpot responses in recovery: Non-DM vs DM across disease severity
Spike
M+N
ORFs(3,6-8)
IFN-γ SFU/million PBMC
400
200
0
-200
(123) n=15
(136) n=6
(75) n=15
(103) n=6
(18) n=15
(9) n=6
(178) n=17
(200) n=11
(150) n=17
(65) n=11
(35) n=17
(23) n=11
C
T cell proliferation in recovery: Non-DM vs DM across disease severity
CD4+ T cells
S1
S2
M
N
ORF3&6
ORF7&8
% proliferation in CD4+ T cells
75
50
25
0
-25
P = 0.0459
P = 0.0386
(15) n=14
(38) n=6
(18) n=14
(27) n=6
(26) n=14
(30) n=6
(13) n=14
(24) n=6
(1) n=14
(7) n=6
(1) n=14
(4) n=6
(13) n=19
(44) n=12
(10) n=19
(27) n=12
(11) n=19
(28) n=12
(9) n=19
(21) n=12
(3) n=19
(9) n=12
(1) n=19
(3) n=12
CD8+ T cells
% proliferation in CD8+ T cells
(3) n=14
(8) n=6
(5) n=14
(9) n=6
(4) n=14
(9) n=6
(8) n=14
(13) n=6
(0) n=14
(0) n=6
(0) n=14
(1) n=6
(18) n=19
(27) n=12
(17) n=19
(16) n=12
(13) n=19
(13) n=12
(18) n=19
(22) n=12
(2) n=19
(2) n=12
(1) n=19
(2) n=12

Figure S7: Antibody and T cell responses in individuals with and without diabetes following recovery from mild-moderate and severe disease

(A) Comparison of SARS-CoV-2 spike, RBD, N -specific IgG responses and neutralising antibody titers in non-diabetic (non-DM, HbA1c < 6.5%) and diabetic (DM, HbA1c ≥ 6.5%) individuals, who recovered from mild-moderate and severe disease. IgG responses and neutralising antibody titers are measured by multiplexed MSD immunoassays and Focus reduction neutralisation (FRNT) assay, respectively, and data are shown in arbitrary units (AU)/mL and IC50, respectively.

(B) Comparison of IFN-γ ELISpot responses to SARS-CoV-2 spike (summed responses to S1 and S2 peptide pools), M+N (summed responses to M and N pools), and ORFs (summed responses to ORF3, 6-8) from cryopreserved PBMCs in recovered individuals with or without diabetes, following recovery from mild-moderate and severe disease. Data are shown in IFN-γ spot-forming units (SFU)/million PBMC.

(C) Comparison of the relative frequency of CD4+ (top panels) and CD8+ (bottom panels) T cells proliferating to individual peptide pools S1, S2, M, N, ORF3&6, ORF7&8, assessed by flow cytometry (gating strategy shown in figure S2A) from cryopreserved PBMC, in SARS-CoV-2 individuals with or without diabetes following recovery from mild-moderate and severe disease.

A two-tailed Wilcoxon rank-sum test was used to compare between the groups (without correction for multiple testing), and the p values are shown on the top of the dot plots, in case of significant differences. The number of individuals (n) evaluated per assay is displayed at the bottom of the corresponding dot plots. Horizontal bars represent the medians, and the median values are shown in brackets immediately above the number of individuals (n) in each column.

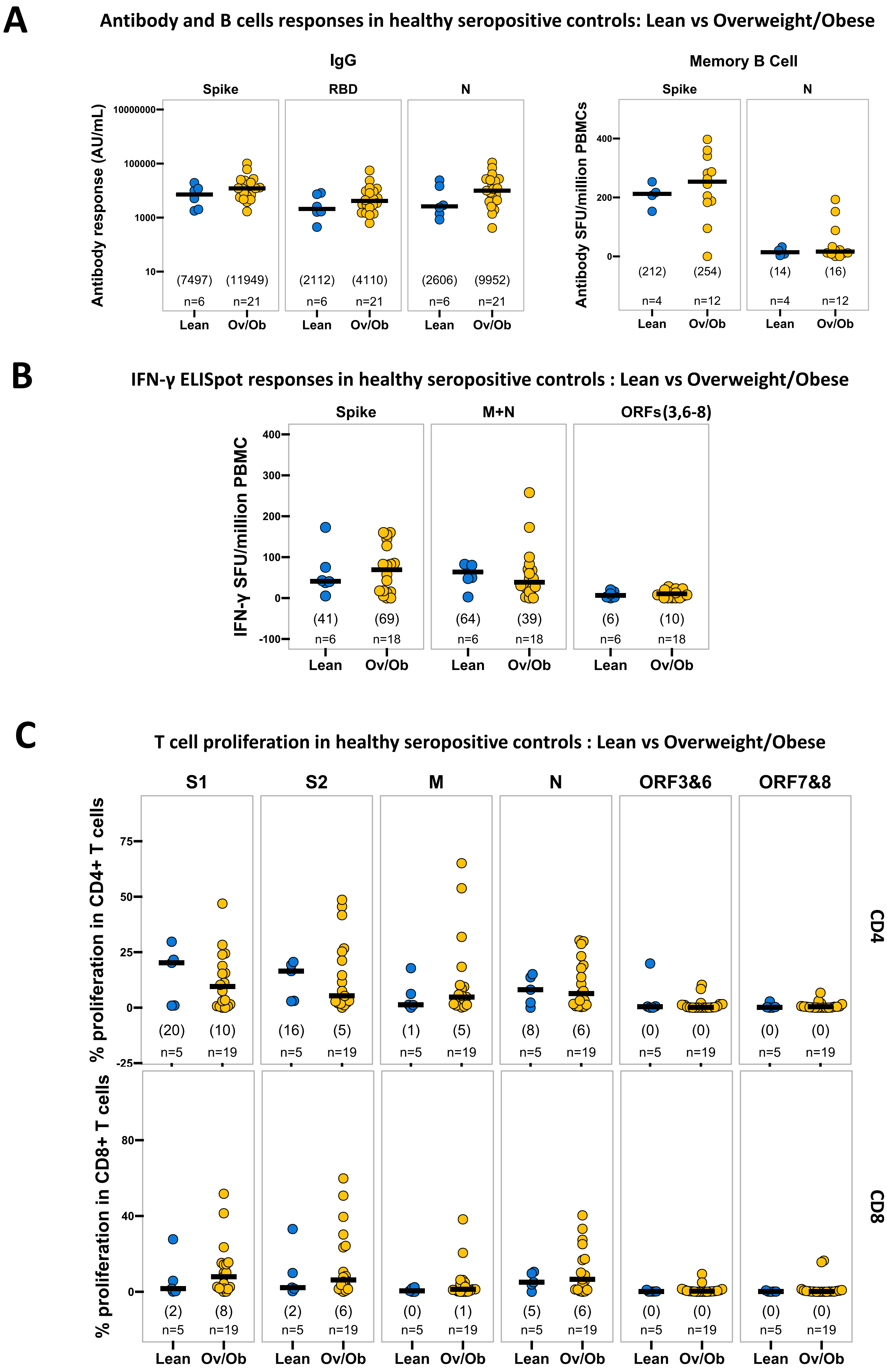
A
Antibody and B cells responses in healthy seropositive controls: Lean vs Overweight/Obese
IgG
Memory B Cell
Spike
RBD
N
Antibody response (AU/mL)
Antibody SFU/million PBMCs
(7497) (11949) (2112) (4110) (2606) (9952)
(212) (254) (14) (16)
n=6 n=21
n=4 n=12
Lean Ov/Ob
B
IFN-γ ELISpot responses in healthy seropositive controls : Lean vs Overweight/Obese
Spike
M+N
ORFs(3,6-8)
IFN-γ SFU/million PBMC
(41) (69) (64) (39) (6) (10)
n=6 n=18
C
T cell proliferation in healthy seropositive controls : Lean vs Overweight/Obese
S1
S2
M
N
ORF3&6
ORF7&8
% proliferation in CD4+ T cells
% proliferation in CD8+ T cells
(20) (10) (16) (5) (1) (5) (8) (6) (0) (0) (0) (0)
(2) (8) (2) (6) (0) (1) (5) (6) (0) (0) (0) (0)
n=5 n=19
CD4
CD8

Figure S8: Antibody and T cell responses are similar among healthy seropositive individuals with and without overweight/obesity.

(A) Comparison of SARS-CoV-2 spike, RBD, N -specific IgG responses, and spike, N -specific memory B cells in lean (BMI = 18.5 – 22.9 kg/m^2) and overweight/obese (BMI ≥ 23 kg/m^2) healthy seropositive individuals. IgG responses, and memory B cell responses are measured by multiplexed MSD immunoassays, and B cell ELISpot assay, respectively, and data are shown in arbitrary units (AU)/mL, and antibody spot-forming units (SFU)/million PBMC respectively.

(B) Comparison of IFN-γ ELISpot responses to SARS-CoV-2 spike (summed responses to S1 and S2 peptide pools), M+N (summed responses to M and N pools), and ORFs (summed responses to ORF3, 6-8) from cryopreserved PBMCs in lean (BMI = 18.5 – 22.9 kg/m^2) and overweight/obese (BMI ≥ 23 kg/m^2) healthy seropositive individuals. Data are shown in IFN-γ spot-forming units (SFU)/million PBMC.

(C) Comparison of the relative frequency of $CD4^+$ (top panels) and $CD8^+$ (bottom panels) T cells proliferating to individual peptide pools S1, S2, M, N, ORF3&6, ORF7&8, assessed by flow cytometry (gating strategy shown in figure S2A) from cryopreserved PBMC, in lean (BMI = 18.5 – 22.9 kg/m^2) and overweight/obese (BMI ≥ 23 kg/m^2) healthy seropositive individuals.

A two-tailed Wilcoxon rank-sum test was used to compare between the groups (without correction for multiple testing), and the p values are shown on the top of the dot plots, in case of significant differences. The number of individuals (n) evaluated per assay is displayed at the bottom of the corresponding dot plots. Horizontal bars represent the medians, and the median values are shown in brackets immediately above the number of individuals (n) in each column.

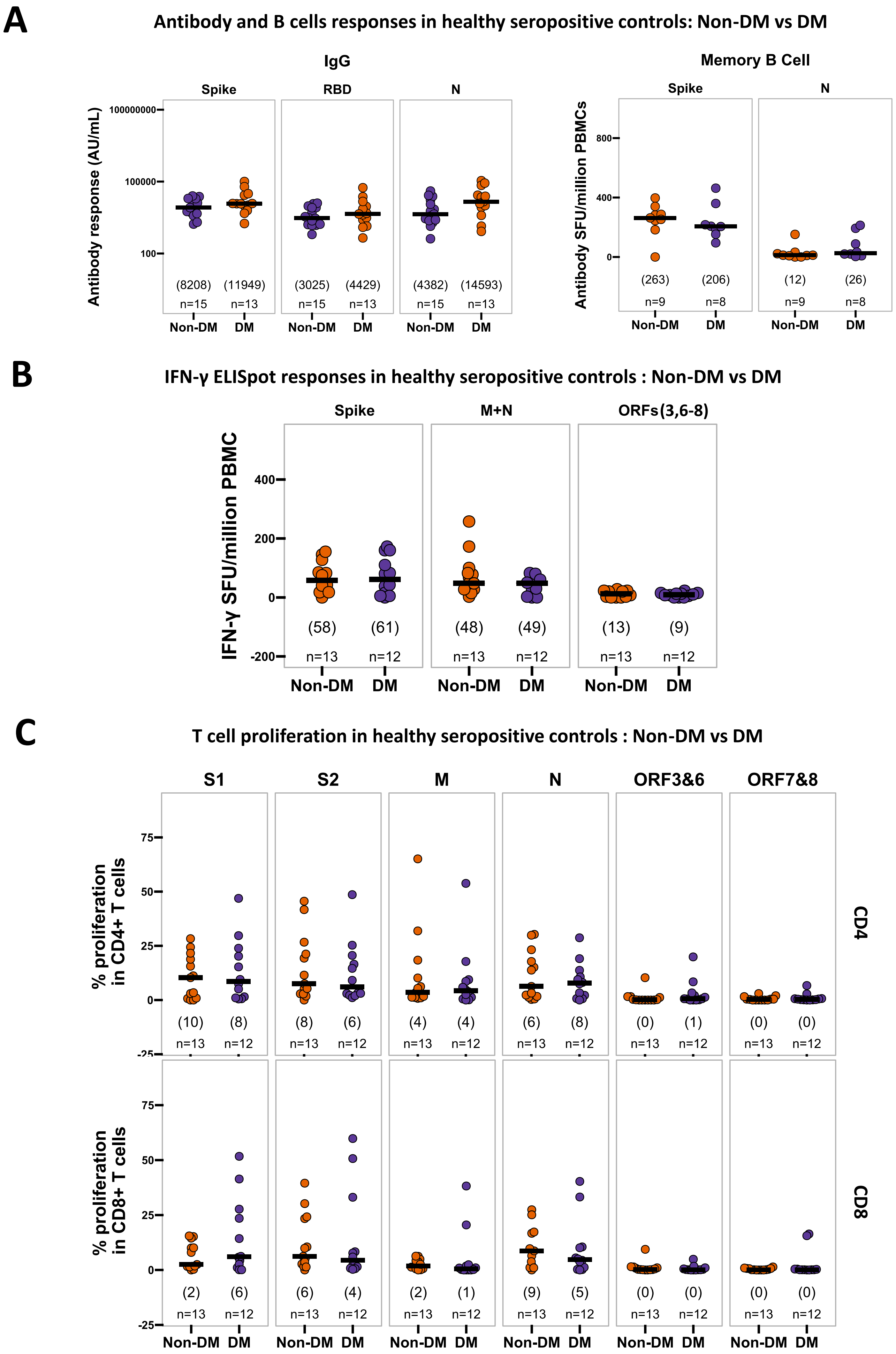

A
Antibody and B cells responses in healthy seropositive controls: Non-DM vs DM
IgG
Spike
RBD
N
Antibody response (AU/mL)
100000000
100000
100
(8208) (11949)
n=15 n=13
(3025) (4429)
n=15 n=13
(4382) (14593)
n=15 n=13
Non-DM DM
Memory B Cell
Spike
N
Antibody SFU/million PBMCs
800
400
0
(263) (206)
n=9 n=8
(12) (26)
n=9 n=8
B
IFN-γ ELISpot responses in healthy seropositive controls : Non-DM vs DM
Spike
M+N
ORFs(3,6-8)
IFN-γ SFU/million PBMC
400
200
0
-200
(58) (61)
(48) (49)
(13) (9)
n=13 n=12
Non-DM DM
C
T cell proliferation in healthy seropositive controls : Non-DM vs DM
S1
S2
M
N
ORF3&6
ORF7&8
% proliferation in CD4+ T cells
75
50
25
0
-25
(10) (8)
(8) (6)
(4) (4)
(6) (8)
(0) (1)
(0) (0)
n=13 n=12
CD4
% proliferation in CD8+ T cells
(2) (6)
(6) (4)
(2) (1)
(9) (5)
(0) (0)
(0) (0)
CD8
Non-DM DM

Figure S9: Antibody and T cell responses are similar among healthy seropositive individuals with and without diabetes.

(A) Comparison of SARS-CoV-2 spike, RBD, N -specific IgG responses, and spike, N -specific memory B cells in non-diabetic (non-DM, HbA1c < 6.5%) and diabetic (DM, HbA1c ≥ 6.5%) healthy seropositive individuals. IgG responses, and memory B cell responses are measured by multiplexed MSD immunoassays, and B cell ELISpot assay, respectively, and data are shown in arbitrary units (AU)/mL, and antibody spot-forming units (SFU)/million PBMC respectively.

(B) Comparison of IFN-γ ELISpot responses to SARS-CoV-2 spike (summed responses to S1 and S2 peptide pools), M+N (summed responses to M and N pools), and ORFs (summed responses to ORF3, 6-8) from cryopreserved PBMCs in healthy seropositive individuals with or without diabetes. Data are shown in IFN-γ spot-forming units (SFU)/million PBMC.

(C) Comparison of the relative frequency of $CD4^+$ (top panels) and $CD8^+$ (bottom panels) T cells proliferating to individual peptide pools S1, S2, M, N, ORF3&6, ORF7&8, assessed by flow cytometry (gating strategy shown in figure S2A) from cryopreserved PBMC, in healthy seropositive individuals with or without diabetes.

A two-tailed Wilcoxon rank-sum test was used to compare between the groups (without correction for multiple testing), and the p values are shown on the top of the dot plots, in case of significant differences. The number of individuals (n) evaluated per assay is displayed at the bottom of the corresponding dot plots. Horizontal bars represent the medians, and the median values are shown in brackets immediately above the number of individuals (n) in each column.

Table S1a. Demographic characteristics of recovered participants with or without overweight/obesity across different assays to measure antibody and B cell responses

Variable	MSD IgG binding assay			Focus reduction neutralisation assay			B cell ELISpot assay		
	**Normal weight** (N = 20)	**Overweight/obesity** (N = 52)	**p-value**	**Normal weight** (N = 20)	**Overweight/obesity** (N = 24)	**p-value**	**Normal weight** (N = 5)	**Overweight/obesity** (N = 18)	**p-value**
**Age in years**, median (IQR[#])	55 (43, 61)	43 (34, 53)	0.020 [a]	55 (43, 61)	43 (35, 51)	0.050 [a]	50 (44, 52)	46 (39, 51)	0.7 [a]
**Sex**			>0.9 [b]			0.7 [b]			0.3 [b]
Female, n (%)	3 (15%)	8 (15%)		3 (15%)	5 (21%)		3 (60%)	5 (28%)	
Male, n (%)	17 (85%)	44 (85%)		17 (85%)	19 (79%)		2 (40%)	13 (72%)	
**Diabetes status**			0.082 [b]			0.083 [b]			>0.9 [b]
Non-diabetic, n (%)	9 (45%)	35 (67%)		9 (45%)	17 (71%)		2 (40%)	9 (50%)	
Diabetic, n (%)	11 (55%)	17 (33%)		11 (55%)	7 (29%)		3 (60%)	9 (50%)	
**Disease severity**			0.3 [b]			0.2 [b]			0.6 [b]
Mild/moderate, n (%)	6 (30%)	22 (42%)		6 (30%)	12 (50%)		3 (60%)	8 (44%)	
Severe, n (%)	14 (70%)	30 (58%)		14 (70%)	12 (50%)		2 (40%)	10 (56%)	
**Days post symptom onset**, median (IQR[#])	54 (36, 110)	59 (38, 100)	>0.9 [a]	54 (36, 110)	47 (34, 88)	0.6 [a]	54 (32, 138)	43 (33, 68)	0.6 [a]

[#]IQR (Interquartile range)
[a] Wilcoxon rank sum test; [b] Pearson's Chi-squared test

Table S1b. Demographic characteristics of recovered participants with or without overweight/obesity across different assays to measure T cell responses

Variable	IFN-γ ELISpot assay			T cell proliferation assay			Intracellular cytokine stimulation assay		
	**Normal weight** (N = 12)	**Overweight/obesity** (N = 36)	**p-value**	**Normal weight** (N = 12)	**Overweight/obesity** (N = 38)	**p-value**	**Normal weight** (N = 4)	**Overweight/obesity** (N = 13)	**p-value**
**Age in years**, median (IQR[#])	53 (43, 55)	44 (37, 52)	0.12 [a]	53 (43, 55)	44 (36, 52)	0.12 [a]	51 (46, 53)	42 (38, 52)	0.8 [a]
**Sex**			0.7 [b]			0.7 [b]			0.3 [b]
Female, n (%)	3 (25%)	6 (17%)		3 (25%)	7 (18%)		3 (75%)	5 (38%)	
Male, n (%)	9 (75%)	30 (83%)		9 (75%)	31 (82%)		1 (25%)	8 (62%)	
**Diabetes status**			0.3 [b]			0.3 [b]			0.6 [b]
Non-diabetic, n (%)	6 (50%)	25 (69%)		6 (50%)	26 (68%)		1 (25%)	7 (54%)	
Diabetic, n (%)	6 (50%)	11 (31%)		6 (50%)	12 (32%)		3 (75%)	6 (46%)	
**Disease severity**			>0.9 [b]			>0.9 [b]			>0.9 [b]
Mild/moderate, n (%)	5 (42%)	15 (42%)		5 (42%)	14 (37%)		2 (50%)	7 (54%)	
Severe, n (%)	7 (58%)	21 (58%)		7 (58%)	24 (63%)		2 (50%)	6 (46%)	
**Days post symptom onset**, median (IQR[#])	54 (38, 114)	55 (35, 91)	0.6 [a]	54 (38, 114)	61 (37, 93)	0.8 [a]	43 (31, 78)	40 (32, 82)	>0.9 [a]

[#]IQR (Interquartile range)
[a] Wilcoxon rank sum test; [b] Pearson's Chi-squared test

Table S1c. Demographic characteristics of recovered participants with or without diabetes across different assays to measure antibody and B cell responses

Variable	MSD IgG binding assay			Focus reduction neutralisation assay			B cell ELISpot assay		
	Non-diabetic (N = 46)	Diabetic (N = 29)	p-value	Non-diabetic (N = 26)	Diabetic (N = 18)	p-value	Non-diabetic (N = 12)	Diabetic (N = 12)	p-value
**Age in years**, median (IQR#)	40 (31, 51)	53 (45, 63)	<0.001 [a]	41 (34, 51)	55 (51, 64)	0.004 [a]	46 (40, 51)	48 (38, 52)	0.7 [a]
**Sex**			>0.9 [b]			>0.9 [b]			>0.9 [b]
Female, n (%)	7 (15%)	4 (14%)		5 (19%)	3 (17%)		4 (33%)	4 (33%)	
Male, n (%)	39 (85%)	25 (86%)		21 (81%)	15 (83%)		8 (67%)	8 (67%)	
**Obesity category**			0.21 [b]			0.083 [b]			>0.9 [b]
Lean, n (%)	9 (20%)	11 (38%)		9 (35%)	11 (61%)		2 (18%)	3 (25%)	
Overweight/obesity, n (%)	35 (76%)	17 (59%)		17 (65%)	7 (39%)		9 (82%)	9 (75%)	
Data missing, n(%)	2 (4%)	1 (3%)					1	0	
**Disease severity**			0.016 [b]			0.4 [b]			>0.9 [b]
Mild/moderate, n (%)	24 (52%)	7 (24%)		12 (46%)	6 (33%)		6 (50%)	6 (50%)	
Severe, n (%)	22 (48%)	22 (76%)		14 (54%)	12 (67%)		6 (50%)	6 (50%)	
**Days post symptom onset**, median (IQR#)	69 (33, 120)	52 (40, 70)	0.3 [a]	70 (33, 120)	48 (36, 65)	0.2 [a]	38 (30, 124)	47 (39, 60)	>0.9 [a]

#IQR (Interquartile range)

[a] Wilcoxon rank sum test; [b] Pearson's Chi-squared test

Table S1d. Demographic characteristics of recovered participants with or without diabetes across different assays to measure T cell responses

Variable	IFN-γ ELISpot assay			T cell proliferation assay			Intracellular cytokine stimulation assay		
	Non-diabetic (N = 32)	Diabetic (N = 17)	p-value	Non-diabetic (N = 33)	Diabetic (N = 18)	p-value	Non-diabetic (N = 9)	Diabetic (N = 9)	p-value
**Age in years**, median (IQR#)	43 (35, 51)	52 (42, 55)	0.11 [a]	44 (34, 52)	52 (39, 55)	0.2 [a]	42 (35, 51)	50 (38, 52)	0.6 [a]
**Sex**			0.7 [b]			0.7 [b]			>0.9 [b]
Female, n (%)	5 (16%)	4 (24%)		6 (18%)	4 (22%)		4 (44%)	4 (44%)	
Male, n (%)	27 (84%)	13 (76%)		27 (82%)	14 (78%)		5 (56%)	5 (56%)	
**Obesity category**			0.3 [b]			0.3 [b]			0.6 [b]
Lean, n (%)	6 (19%)	6 (35%)		6 (19%)	6 (33%)		1 (13%)	3 (33%)	
Overweight/obesity, n (%)	25 (81%)	11 (65%)		26 (81%)	12 (67%)		7 (88%)	6 (67%)	
Data missing, n(%)	1	0		1	0		1	0	
**Disease severity**			0.4 [b]			0.5 [b]			>0.9 [b]
Mild/moderate, n (%)	15 (47%)	6 (35%)		14 (42%)	6 (33%)		5 (56%)	5 (56%)	
Severe, n (%)	17 (53%)	11 (65%)		19 (58%)	12 (67%)		4 (44%)	4 (44%)	
**Days post symptom onset**, median (IQR#)	69 (34, 119)	51 (40, 60)	0.3 [a]	76 (36, 119)	52 (41, 65)	0.2 [a]	35 (29, 123)	43 (35, 54)	0.8 [a]

#IQR (Interquartile range)

[a] Wilcoxon rank sum test; [b] Pearson's Chi-squared test

Table S2a. Generalised linear regression models of antibody (IgG, neutralising Ab) and memory B cell responses in SARS-CoV-2 recovered patients

	Anti-spike IgG			Anti-RBD IgG			Anti-N IgG			Neutralising antibody			Anti-spike memory B cells			Anti-N memory B cells		
**Coefficient**	**Est**	**SE**	**p value**	**Est**	**SE**	**p value**	**Est**	**SE**	**p value**	**Est**	**SE**	**p value**	**Est**	**SE**	**p value**	**Est**	**SE**	**p value**
**Age**	0.2615	0.1316	0.0512	0.2219	0.1319	0.0974	0.1134	0.1421	0.4278	0.2088	0.1794	0.2518	0.2054	0.2667	0.4523	0.1126	0.2476	0.6553
**Sex (male)**	-0.2075	0.2742	0.4519	-0.1770	0.2748	0.5219	-0.1592	0.2960	0.5925	-0.1880	0.3405	0.5843	0.2552	0.5673	0.6589	-0.0994	0.5266	0.8526
**DM (yes)**	0.1133	0.2226	0.6126	0.1848	0.2231	0.4105	0.0159	0.2403	0.9474	-0.2130	0.3030	0.4864	-0.3309	0.4765	0.4973	0.0078	0.4423	0.9862
**Obesity (yes)**	0.1981	0.2264	0.3846	0.1861	0.2269	0.4152	0.3610	0.2444	0.1445	-0.7077	0.2788	**0.0155**	-0.2757	0.5925	0.6481	-0.6133	0.5501	0.2813
**Disease Severity (severe)**	0.7628	0.2464	**0.0029**	0.7212	0.2470	**0.0048**	0.8410	0.2660	**0.0024**	0.6099	0.3077	0.0549	0.1470	0.5161	0.7794	-0.0677	0.4791	0.8895
**Days pso**	-0.1537	0.1050	0.1482	-0.2229	0.1053	**0.0381**	-0.1740	0.1134	0.1296	-0.1988	0.1424	0.1711	0.2341	0.2735	0.4047	0.3316	0.2539	0.2100

Est (estimates), SE (standard error), pso (post symptom onset)

Table S2b. Generalised linear regression models of IFN-γ T cell ELISpot responses in SARS-CoV-2 recovered patients

	S1 IFN-γ ELISpot			S2 IFN-γ ELISpot			M IFN-γ ELISpot			N IFN-γ ELISpot			ORF3+6 IFN-γ ELISpot			ORF7+8 IFN-γ ELISpot		
**Coefficient**	**Est**	**SE**	**p value**	**Est**	**SE**	**p value**	**Est**	**SE**	**p value**	**Est**	**SE**	**p value**	**Est**	**SE**	**p value**	**Est**	**SE**	**p value**
**Age**	0.2439	0.1633	0.1430	0.3061	0.1728	0.0839	0.1504	0.1691	0.3788	0.1408	0.1752	0.4263	0.1169	0.1730	0.5031	0.1425	0.1779	0.4275
**Sex (male)**	-0.2320	0.3547	0.5168	0.0767	0.3752	0.8391	-0.2778	0.3672	0.4536	-0.2894	0.3806	0.4513	-0.3771	0.3758	0.3215	-0.0962	0.3863	0.8046
**DM (yes)**	0.2647	0.3047	0.3900	-0.1007	0.3223	0.7563	-0.0012	0.3154	0.9971	0.1480	0.3269	0.6532	-0.3354	0.3228	0.3049	-0.4161	0.3319	0.2170
**Obesity (yes)**	0.6928	0.3229	**0.0379**	0.4721	0.3416	0.1745	0.6411	0.3343	0.0621	0.5200	0.3465	0.1410	0.3884	0.3421	0.2628	-0.0978	0.3517	0.7823
**Disease Severity (severe)**	0.3741	0.3112	0.2362	0.2880	0.3292	0.3867	0.4061	0.3221	0.2145	0.2215	0.3339	0.5107	0.4818	0.3297	0.1515	0.4011	0.3389	0.2435
**Days pso**	-0.1033	0.1446	0.4791	0.0355	0.1530	0.8179	0.1950	0.1497	0.2001	0.2164	0.1552	0.1706	-0.1146	0.1532	0.4589	0.0359	0.1575	0.8207

Est (estimates), SE (standard error), pso (post symptom onset)

Table S2c. Generalised linear regression models of CD4 T cell proliferative responses in SARS-CoV-2 recovered patients

	**S1 CD4 proliferation**			**S2 CD4 proliferation**			**M CD4 proliferation**			**N CD4 proliferation**			**ORF3+6 CD4 proliferation**			**ORF7+8 CD4 proliferation**		
**Coefficient**	**Est**	**SE**	**p value**	**Est**	**SE**	**p value**	**Est**	**SE**	**p value**	**Est**	**SE**	**p value**	**Est**	**SE**	**p value**	**Est**	**SE**	**p value**
**Age**	0.2340	0.1598	0.1504	0.2142	0.1637	0.1978	0.0762	0.1660	0.6486	0.1505	0.1671	0.3727	0.3135	0.1580	0.0536	0.4431	0.1437	**0.0036**
**Sex (male)**	-0.2905	0.3594	0.4235	-0.1127	0.3682	0.7610	-0.2992	0.3734	0.4274	-0.2926	0.3758	0.4405	0.1262	0.3553	0.7242	0.5546	0.3231	0.0933
**DM (yes)**	0.5313	0.3147	0.0986	0.5313	0.3224	0.1067	0.5095	0.3270	0.1266	0.3326	0.3291	0.3178	0.3569	0.3112	0.2577	0.5901	0.2830	**0.0430**
**Obesity (yes)**	0.0663	0.3406	0.8465	0.1532	0.3489	0.6627	0.0884	0.3539	0.8039	0.1225	0.3561	0.7325	0.0989	0.3367	0.7703	0.2534	0.3062	0.4124
**Disease Severity (severe)**	-0.0093	0.3172	0.9767	-0.0542	0.3249	0.8684	-0.0908	0.3296	0.7842	0.0633	0.3317	0.8496	0.2303	0.3136	0.4668	-0.0186	0.2852	0.9483
**Days pso**	0.0012	0.1534	0.9936	0.0623	0.1571	0.6935	-0.0291	0.1593	0.8558	0.0033	0.1603	0.9839	0.0507	0.1516	0.7398	0.0141	0.1379	0.9193

Est (estimates), SE (standard error), pso (post symptom onset)

Table S2d. Generalised linear regression models of CD8 T cell proliferative responses in SARS-CoV-2 recovered patients

	**S1 CD8 proliferation**			**S2 CD8 proliferation**			**M CD8 proliferation**			**N CD8 proliferation**			**ORF3+6 CD8 proliferation**			**ORF7+8 CD8 proliferation**		
**Coefficient**	**Est**	**SE**	**p value**	**Est**	**SE**	**p value**	**Est**	**SE**	**p value**	**Est**	**SE**	**p value**	**Est**	**SE**	**p value**	**Est**	**SE**	**p value**
**Age**	0.2227	0.1469	0.1368	0.2834	0.1473	0.0611	0.1215	0.1627	0.4593	0.1715	0.1595	0.2881	0.2439	0.1588	0.1320	0.4568	0.1356	**0.0016**
**Sex (male)**	-0.3662	0.3304	0.2738	-0.4932	0.3314	0.1440	-0.3724	0.3659	0.3145	-0.1622	0.3587	0.6534	-0.2693	0.3573	0.4551	0.1134	0.3049	0.7118
**DM (yes)**	0.4680	0.2893	0.1131	0.3532	0.2902	0.2301	0.3330	0.3204	0.3044	0.2438	0.3141	0.4419	0.0904	0.3128	0.7740	0.3822	0.2670	0.1595
**Obesity (yes)**	0.6695	0.3131	**0.0382**	0.8002	0.3140	**0.0145**	0.5399	0.3467	0.1268	0.4656	0.3399	0.1779	0.4066	0.3386	0.2364	0.8032	0.2889	**0.0080**
**Disease Severity (severe)**	0.4684	0.2916	0.1155	0.2513	0.2924	0.3950	0.1253	0.3229	0.6999	0.4430	0.3166	0.1689	0.3634	0.3153	0.2554	0.3205	0.2691	0.2402
**Days pso**	0.0863	0.1410	0.5436	0.0462	0.1414	0.7453	0.1522	0.1561	0.3351	0.0701	0.1531	0.6491	0.0480	0.1524	0.7542	0.0118	0.1301	0.9282

Est (estimates), SE (standard error), pso (post symptom onset)

Table S2e. Generalised linear regression models of CD4 T cell cytokine responses in SARS-CoV-2 recovered patients

	CD4 Spike IFN-γ			CD4 Spike IL-2			CD4 Spike TNF			CD4 M+N IFN-γ			CD4 M+N IL-2			CD4 M+N TNF		
**Coefficient**	**Est**	**SE**	**p value**	**Est**	**SE**	**p value**	**Est**	**SE**	**p value**	**Est**	**SE**	**p value**	**Est**	**SE**	**p value**	**Est**	**SE**	**p value**
**Age**	0.3170	0.2982	0.3128	0.2555	0.2459	0.3233	0.2092	0.3048	0.5081	0.2674	0.3133	0.4135	0.1944	0.2594	0.4708	-0.3688	0.3110	0.2632
**Sex (male)**	0.3723	0.6867	0.5996	1.0306	0.5663	0.0988	0.3834	0.7020	0.5970	0.2759	0.7215	0.7102	0.5908	0.5973	0.3460	-0.2361	0.7162	0.7485
**DM (yes)**	-0.7169	0.5252	0.2022	-0.4192	0.4331	0.3560	-0.4518	0.5369	0.4198	-0.7481	0.5518	0.2050	-0.3923	0.4568	0.4106	0.0420	0.5478	0.9403
**Obesity (yes)**	0.1716	0.6298	0.7908	0.1389	0.5193	0.7945	0.8563	0.6438	0.2130	0.5275	0.6617	0.4438	-0.0329	0.5478	0.9533	-0.1901	0.6568	0.7782
**Disease Severity (severe)**	0.3686	0.5900	0.5462	0.1065	0.4865	0.8312	-0.4126	0.6031	0.5094	-0.1854	0.6199	0.7710	0.2750	0.5132	0.6037	-0.1665	0.6154	0.7922
**Days pso**	-0.2078	0.3253	0.5373	-0.3230	0.2683	0.2562	0.0027	0.3325	0.9936	-0.1130	0.3418	0.7478	-0.5099	0.2830	0.1017	-0.4725	0.3393	0.1939

Est (estimates), SE (standard error), pso (post symptom onset)

Table S2f. Generalised linear regression models of CD8 T cell cytokine responses in SARS-CoV-2 recovered patients

	CD8 Spike IFN-γ			CD8 Spike IL-2			CD8 Spike TNF			CD8 M+N IFN-γ			CD8 M+N IL-2			CD8 M+N TNF		
**Coefficient**	**Est**	**SE**	**p value**	**Est**	**SE**	**p value**	**Est**	**SE**	**p value**	**Est**	**SE**	**p value**	**Est**	**SE**	**p value**	**Est**	**SE**	**p value**
**Age**	-0.3468	0.3103	0.2899	-0.5560	0.2141	**0.0266**	-0.2412	0.3162	0.4633	-0.0623	0.3305	0.8542	-0.5717	0.2919	0.0786	-0.2982	0.2994	0.3427
**Sex (male)**	-0.4403	0.7146	0.5516	0.1160	0.4930	0.8187	-0.6419	0.7282	0.3987	-0.1148	0.7610	0.8831	0.0510	0.6722	0.9410	0.3871	0.6893	0.5868
**DM (yes)**	-0.6703	0.5465	0.2481	-0.2953	0.3771	0.4516	0.2779	0.5569	0.6286	-0.0871	0.5820	0.8840	-0.1144	0.5141	0.8283	0.0486	0.5272	0.9284
**Obesity (yes)**	-0.0194	0.6553	0.9770	1.2171	0.4521	**0.0226**	0.6295	0.6678	0.3680	0.6008	0.6979	0.4095	0.3194	0.6164	0.6156	0.6140	0.6322	0.3543
**Disease Severity (severe)**	0.6468	0.6140	0.3169	0.6714	0.4236	0.1441	0.6328	0.6256	0.3356	-0.7529	0.6538	0.2763	0.2345	0.5775	0.6933	-0.3396	0.5923	0.5790
**Days pso**	0.0369	0.3385	0.9154	0.3537	0.2335	0.1608	0.2361	0.3449	0.5091	-0.0864	0.3605	0.8155	-0.2600	0.3184	0.4332	0.0121	0.3265	0.9711

Est (estimates), SE (standard error), pso (post symptom onset)

Table S3. Antibodies used for intracellular cytokine staining and proliferation assay

Marker	Fluorochrome	Clone	Isotype	Species reactivity	Host Species	Manufacturer	Catalogue number	Dilution used
CD14	APC-Fire750	M5E2	IgG2a, κ	Human	Mouse	Biolegend, UK	301854	200
CD3	PerCP	UCHT1	IgG1, κ	Human	Mouse	Biolegend, UK	300428	100
CD3	FITC	UCHT1	IgG1, κ	Human	Mouse	Biolegend, UK	300440	50
CD4	APC	RPA-T4	IgG1, κ	Human	Mouse	Biolegend, UK	300514	200
CD8	PE-Cy7	RPA-T8	IgG1, κ	Human	Mouse	Biolegend, UK	301012	200
CD8	BV510	RPA-T8	IgG1, κ	Human	Mouse	Biolegend, UK	301048	600
IFN-γ	PE	4S.B3	IgG1, κ	Human	Mouse	Biolegend, UK	502508	50
IL-2	PE-Cy7	MQ1-17H12	IgG2a, κ	Human	Rat	eBioscience, USA	25-7029-41	100
TNF	FITC	MAb11	IgG1, κ	Human	Mouse	Biolegend, UK	502906	40

Table S4a. Demographic characteristics of participants selected for Focus Reduction Neutralisation assay

**Variable**	**Recovered (Mild/moderate)** (N = 18)	**Recovered (Severe)** (N = 26)
**Age in years**, median (IQR)	40 (33, 44)	56 (50, 64)
**Sex**		
Female, n (%)	3 (17%)	5 (19%)
Male, n (%)	15 (83%)	21 (81%)
**Diabetes status**		
Non-diabetic, n (%)	12 (67%)	14 (54%)
Diabetic, n (%)	6 (33%)	12 (46%)
**Obesity category**		
Normal weight, n (%)	6 (33%)	14 (54%)
Overweight/obesity, n (%)	12 (67%)	12 (46%)
Data missing, n (%)		
**Days post symptom onset**, median (IQR)	47 (35, 120)	52 (34, 77)
IQR (Interquartile range)		

Table S4b. Demographic characteristics of participants selected for B cell ELISpot assay

**Variable**	**Healthy Seronegative** (N = 7)	**Healthy Seropositive** (N = 17)	**Recovered (Mild/moderate)** (N = 12)	**Recovered (Severe)** (N = 12)
**Age in years**, median (IQR)	52 (43, 54)	41 (36, 48)	42 (34, 47)	51 (46, 51)
**Sex**				
Female, n (%)	4 (57%)	8 (47%)	3 (25%)	5 (42%)
Male, n (%)	3 (43%)	9 (53%)	9 (75%)	7 (58%)
**Diabetes status**				
Non-diabetic, n (%)	3 (43%)	9 (53%)	6 (50%)	6 (50%)
Diabetic, n (%)	4 (57%)	8 (47%)	6 (50%)	6 (50%)
**Obesity category**				
Normal weight, n (%)	2 (29%)	4 (25%)	3 (27%)	2 (17%)
Overweight/obesity, n (%)	5 (71%)	12 (75%)	8 (73%)	10 (83%)
Data missing, n (%)	0	1	1	0
**Days post symptom onset**, median (IQR)	NA	NA	43 (34, 105)	49 (32, 67)
IQR (Interquartile range)				

Table S4c. Demographic characteristics of participants selected for IFN-γ ELISpot assay

**Variable**	**Healthy Seronegative** (N = 23)	**Healthy Seropositive** (N = 25)	**Recovered (Mild/moderate)** (N = 21)	**Recovered (Severe)** (N = 28)
**Age in years**, median (IQR)	44 (39, 53)	39 (33, 45)	39 (33, 44)	51 (44, 57)
**Sex**				
Female, n (%)	8 (35%)	8 (32%)	3 (14%)	6 (21%)
Male, n (%)	15 (65%)	17 (68%)	18 (86%)	22 (79%)
**Diabetes status**				
Non-diabetic, n (%)	17 (74%)	13 (52%)	15 (71%)	17 (61%)
Diabetic, n (%)	6 (26%)	12 (48%)	6 (29%)	11 (39%)
**Obesity category**				
Normal weight, n (%)	6 (27%)	6 (25%)	5 (25%)	7 (25%)
Overweight/obesity, n (%)	16 (73%)	18 (75%)	15 (75%)	21 (75%)
Data missing, n (%)	1	1	1	0
**Days post symptom onset**, median (IQR)	NA	NA	42 (33, 118)	59 (38, 91)
IQR (Interquartile range)				

Table S4d. Demographic characteristics of participants selected for T cell Proliferation assay

**Variable**	**Healthy Seronegative** (N = 24)	**Healthy Seropositive** (N = 25)	**Recovered (Mild/moderate)** (N = 20)	**Recovered (Severe)** (N = 31)
**Age in years**, median (IQR)	43 (38, 52)	39 (35, 45)	40 (33, 44)	51 (41, 57)
**Sex**				
Female, n (%)	8 (33%)	8 (32%)	3 (15%)	7 (23%)
Male, n (%)	16 (67%)	17 (68%)	17 (85%)	24 (77%)
**Diabetes status**				
Non-diabetic, n (%)	18 (75%)	13 (52%)	14 (70%)	19 (61%)
Diabetic, n (%)	6 (25%)	12 (48%)	6 (30%)	12 (39%)
**Obesity category**				
Normal weight, n (%)	6 (26%)	5 (21%)	5 (26%)	7 (23%)
Overweight/obesity, n (%)	17 (74%)	19 (79%)	14 (74%)	24 (77%)
Data missing, n (%)	1	1	1	0
**Days post symptom onset**, median (IQR)	NA	NA	43 (34, 119)	61 (41, 92)
IQR (Interquartile range)				

Table S4e. Demographic characteristics of participants selected for Intracellular Cytokine Stimulation assay

**Variable**	**Healthy Seronegative** (N = 5)	**Healthy Seropositive** (N = 13)	**Recovered (Mild/moderate)** (N = 10)	**Recovered (Severe)** (N = 8)
**Age in years**, median (IQR)	53 (50, 54)	41 (35, 45)	39 (33, 50)	51 (48, 56)
**Sex**				
Female, n (%)	4 (80%)	8 (62%)	3 (30%)	5 (63%)
Male, n (%)	1 (20%)	5 (38%)	7 (70%)	3 (38%)
**Diabetes status**				
Non-diabetic, n (%)	2 (40%)	7 (54%)	5 (50%)	4 (50%)
Diabetic, n (%)	3 (60%)	6 (46%)	5 (50%)	4 (50%)
**Obesity category**				
Normal weight, n (%)	2 (40%)	3 (23%)	2 (22%)	2 (25%)
Overweight/obesity, n (%)	3 (60%)	10 (77%)	7 (78%)	6 (75%)
Data missing, n (%)	0	0	1	0
**Days post symptom onset**, median (IQR)	NA (NA, NA)	NA (NA, NA)	40 (32, 82)	41 (32, 63)
IQR (Interquartile range)				

Methods

Generalised linear models

Generalised linear models (GLMs) were performed to estimate the association of obesity (categorical) and DM (categorical) with antibody, B cell, and T cell immune responses (all continuous and log-transformed) while adjusted for age (continuous), sex (discrete), disease severity (categorical) and days post symptoms onset (continuous) in SARS-CoV-2 recovered patients. All continuous variables were standardised by z-score normalisation. Normality of the data was tested by Shapiro–Wilk test, histogram, and Q-Q diagnostic plots. Interactions and co-linearity between variables were explored.

Model < - glm (immune response ∼ age + sex + diabetes status + obesity category + disease severity + days post symptoms onset, data = data)
